# Personalized Management of Cytopenias in Chronic Liver Disease: From Pathophysiology to Treatment Strategies

**DOI:** 10.1002/jgh3.70421

**Published:** 2026-05-20

**Authors:** Xiaoxiao Wang, Shuyan Fu, Xiaohong Zhu, Yanping Zhao

**Affiliations:** ^1^ The First Affiliated Hospital of Zhejiang Chinese Medical University (Zhejiang Provincial Hospital of Chinese Medicine) Hangzhou Zhejiang China

**Keywords:** chronic liver disease, leukopenia, pathophysiology, thrombocytopenia, treatment

## Abstract

Thrombocytopenia and leukopenia are among the most common hematological abnormalities in patients with chronic liver disease (CLD), particularly among those with cirrhosis. Thrombocytopenia and leukopenia are associated with increased risks of bleeding, infection, treatment delay, and poor clinical outcomes. Their pathogenesis is multifactorial, comprising hypersplenism‐related sequestration, decreased synthesis of hematopoietic growth factors, such as thrombopoietin, bone marrow suppression, immune‐mediated destruction, and systemic inflammation. This review summarizes the current understanding of the mechanisms underlying cytopenias in CLD and discusses available therapeutic strategies, including platelet transfusion, spleen‐targeted interventions, thrombopoietin‐based treatments, thrombopoietin receptor agonists, and granulocyte colony‐stimulating factor. Particular attention is given to the efficacy and safety profiles of these treatment modalities and their potential risks, such as thromboembolic events due to thrombopoietin receptor agonists. Importantly, effective management of cytopenias in CLD should be tailored based on disease severity, etiology of cirrhosis, dominant pathophysiological mechanisms, thrombotic risk, and the urgency of invasive procedures. Integrating these factors into clinical decision‐making may improve the outcomes of patients and optimize treatment choices. Future studies should focus on risk‐stratified treatment algorithms, long‐term safety of emerging agents, and combination therapeutic strategies.

## 1 Introduction

Cytopenia is a prevalent complication of chronic liver disease (CLD), which affects up to 84% of patients with cirrhosis. Thrombocytopenia is the earliest and most common hematologic abnormality in CLD, occurring in 6%–78% of patients. The prevalence of thrombocytopenia in CLD progressively increases from chronic hepatitis to cirrhosis. Leukopenia affects approximately 42% of patients with cirrhosis, while anemia is detected in nearly 37%. Thrombocytopenia often coexists with leukopenia, and both are associated with increased mortality and a higher risk of transplantation in patients with cirrhosis [1, 2].

Internationally, thrombocytopenia is defined as a platelet count < 150 × 10^9^/L and can be categorized as mild (100–149 × 10^9^/L), moderate (50–99 × 10^9^/L), or severe (< 50 × 10^9^/L) [3]. Mild to moderate thrombocytopenia typically does not affect diagnostic or therapeutic decisions, as patients with mild reductions in platelet count rarely experience spontaneous bleeding. However, severe thrombocytopenia greatly escalates the risk of bleeding during invasive procedures or surgeries, particularly in patients with cirrhosis. This condition can also limit critical treatment options, including targeted therapy, immunotherapy, or chemotherapy [4, 5, 6].

Patients with cirrhosis and leukopenia, especially those with neutropenia, have a significantly increased risk of infections [7]. The severity of neutropenia is classified based on the absolute neutrophil count (ANC): mild: 1.0–1.5 × 10^9^/L; moderate: 0.5–1.0 × 10^9^/L; severe: < 0.5 × 10^9^/L [8].

The mechanisms behind thrombocytopenia and leukopenia in cirrhosis are complex and comprise various factors, including hypersplenism, insufficient production of hematopoietic growth factors, and bone marrow suppression. However, the relative importance of these factors and how they interact in different patients remains incompletely elucidated [9, 10]. Clinical management primarily involves platelet transfusion and spleen‐targeted interventions, which aim to increase peripheral blood cell counts. In recent years, newer targeted therapies, including thrombopoietin receptor agonists (TPO‐RAs) and granulocyte colony‐stimulating factor (G‐CSF), offer new options for cytopenias; however, their efficacy and long‐term safety, suitable patient populations, and optimal timing require further investigation through high‐quality studies.

Formulating management strategies requires integrating several clinical factors, including liver disease severity (Child–Pugh class and model for end‐stage liver disease [MELD] score), cirrhosis etiology, the predominant mechanism causing cytopenia, thrombotic risks, the presence of hepatocellular carcinoma (HCC), and the urgency of invasive procedures. Incorporating these clinical determinants into treatment algorithms may facilitate more rational and individualized decision‐making.

## 2 Pathophysiology

Leukopenia and thrombocytopenia result from multiple common and specific pathophysiological mechanisms. The heterogeneity of these mechanisms directly guides the selection of clinical intervention strategies.

### 2.1 Decreased Production

#### 2.1.1 Reduced Production of Hematopoietic Growth Factors

Thrombopoietin (TPO) plays a crucial role in regulating platelet production. It is primarily synthesized by hepatocytes and exerts its effects by binding to its specific receptor c‐Mpl [11, 12]. TPO binds to the c‐Mpl receptor on the surface of platelets and megakaryocytes, where it is internalized and degraded, forming a negative feedback regulation. Therefore, a lower platelet count is associated with slower TPO degradation, leading to increased TPO concentration that stimulates the bone marrow to generate new platelets [13]. Animal studies support this mechanism: c‐Mpl gene‐deficient mice showed approximately an 85% decrease in platelet and megakaryocyte counts, accompanied by increased TPO concentrations [14, 15]. In cirrhosis, massive hepatocyte loss impairs hepatic TPO synthesis, disrupting the “liver‐bone marrow axis” and interfering with adequate thrombopoietic compensation. This is supported by a higher incidence of thrombocytopenia in cirrhosis (64% vs. 5.5%) and its positive correlation with fibrosis severity [2, 16]. Splenic platelet retention may also exacerbate thrombocytopenia by accelerating TPO‐mediated clearance [17].

Granulocyte production is mainly regulated by G‐CSF and granulocyte/macrophage colony‐stimulating factor (GM‐CSF). Although there are few studies on the effects of cirrhosis on the synthesis of these two endogenous factors, clinical trials provide controversial results. Treatment with exogenous GM‐CSF or G‐CSF significantly elevates neutrophil counts in patients with cirrhosis or liver failure, suggesting a relative deficiency of endogenous hematopoietic factors [18, 19, 20].

#### 2.1.2 Bone Marrow Suppression

The etiology and associated therapeutic approaches for CLD can directly suppress bone marrow, which is a modifiable target. Key contributing factors include medications, viral hepatitis infections, and alcohol‐related damage. Drugs such as antibiotics, azathioprine, and interferon (IFN) can also cause pancytopenia through bone marrow suppression. Bone marrow becomes increasingly sensitive to drug toxicity with the deterioration of liver function. Notably, the risk of leukopenia induced by β‐lactam antibiotics was positively correlated with the severity of liver dysfunction [21]. Azathioprine and IFN are effective treatments for autoimmune hepatitis and viral hepatitis, respectively; however, bone marrow suppression is their common side effect, most notably manifesting as leukopenia [22, 23]. IFN inhibits transcription factors linked to late megakaryocyte maturation, thereby blocking cytoplasmic maturation and thrombopoiesis and resulting in significant thrombocytopenia [24]. IFN‐induced hematopoietic suppression often necessitates dose adjustments or treatment interruption.

Thrombocytopenia is common in patients with hepatitis C virus (HCV) infection, with prevalence rates varying based on disease stage, geography, and inclusion criteria [25, 26]. Studies have shown that hepatitis B core antigen or HCV RNA can be detected in the bone marrow cells of some patients who often exhibit altered bone marrow morphology and lower white blood cell and platelet counts [27, 28, 29]. This suggests possible direct viral suppression, though causal evidence is lacking. Alcoholic patients often show pancytopenia, with bone marrow pathology marked by vacuolation of primitive cells [30]. By suppressing the activity of myeloid progenitor cells and disrupting the bone marrow microenvironment, alcohol and its metabolites can impair granulocyte production, leading to leukopenia [31]. High‐concentration alcohol can shorten platelet lifespan and directly inhibit megakaryocyte proliferation and differentiation. Platelet counts recover after abstinence, suggesting that the inhibitory effect of alcohol on megakaryopoiesis is partly reversible [32, 33].

The core mechanism of thrombocytopenia in cirrhosis is insufficient TPO synthesis due to hepatocyte injury, whereas leukopenia is mainly attributed to bone marrow suppression caused by medications, viral infection, or alcohol. Targeting the underlying etiology (e.g., alcohol abstinence, antiviral therapy, discontinuation of offending drugs) is the key strategy to improve hematological abnormalities.

### 2.2 Increased Destruction/Consumption

#### 2.2.1 Splenomegaly

Portal hypertension caused by cirrhosis can obstruct splenic venous return, causing congestive splenomegaly and hypersplenism. This condition leads to prolonged retention and accelerated destruction of blood cells in splenic sinusoids, decreasing peripheral blood cell counts [34]. Several studies confirmed significant correlations between hepatic venous pressure gradients, spleen volume, and platelet/white blood cell counts [1, 35, 36]. Studies showed that microRNA‐615‐3p, highly expressed in splenic macrophages, enhances phagocytic activity, thereby accelerating platelet clearance [37]. A study reported that partial splenectomy can significantly increase platelet and white blood cell counts in patients with cirrhosis and hypersplenism, confirming the central role of splenomegaly [38].

#### 2.2.2 Other Mechanisms

Cirrhosis‐induced portal hypertension increases shear stress in the vascular system. Under high‐shear conditions, von Willebrand factor (vWF) shifts from a globular to an extended chain state, which more readily binds to platelets and enhances platelet adhesion, aggregation, and clearance [39, 40]. Normally, ADAMTS13 cleaves highly polymerized vWF under shear stress [41, 42]. Declining liver function reduces ADAMTS13 activity, leading to the accumulation of high‐multimer vWF and excessive platelet consumption [43].

Patients with CLD, particularly those with HCV infection, frequently develop antiplatelet antibodies, with a positivity rate reaching 64%–88%. Antibody levels show a significant negative correlation with platelet counts, suggesting that immune‐mediated platelet destruction is one of the pathological mechanisms in patients with thrombocytopenia [44, 45, 46]. Approximately 70% of chronic HCV‐infected individuals develop at least one extrahepatic manifestation, like mixed cryoglobulinemia [47]. Studies have shown that this condition is linked to HCV infection, as cryoglobulins may accelerate platelet clearance by forming immune complexes [48].

The clearance rates of tissue plasminogen activator (t‐PA) and plasminogen activator inhibitor‐1 (PAI‐1) and the synthesis of plasminogen inhibitor α‐2‐antiplasmin and tissue‐acquired fibrinolytic inhibitor (TAFI) are downregulated in patients with cirrhosis. This weakens fibrinolytic capacity, leading to hyperfibrinolysis in approximately 30% of patients and increasing the risk of intravascular coagulation and excessive platelet consumption. This condition suggests a positive correlation with Child–Pugh classification [49, 50].

Due to impaired intestinal barrier function and bacterial translocation, patients with cirrhosis often exhibit elevated levels of endotoxin, even in the absence of overt infection [51]. Patients with cirrhosis and thrombocytopenia exhibit higher levels of endotoxins, with platelet count revealing a negative correlation with endotoxin levels [52]. Endotoxins can induce disseminated intravascular coagulation (DIC), activate platelets and the TLR4 signaling pathway [53], and elevate the risk of thrombosis by inhibiting ADAMTS13 activity [54], collectively exacerbating platelet depletion [42].

Infection, particularly sepsis, is a major cause of thrombocytopenia in cirrhosis. Bacterial infections affect up to 47% of patients with cirrhosis. A study of 304 ICU‐admitted patients with severe sepsis reported a 47.6% incidence of thrombocytopenia [55, 56]. Mechanistically, sepsis‐induced platelet activation enhances adhesion to neutrophils and endothelial cells, leading to aggregation and microcirculatory dysfunction, thereby exacerbating platelet consumption [57].

Splenomegaly and hypersplenism are the major common factors leading to thrombocytopenia and leukopenia. In addition, immune‐mediated destruction, endothelial dysfunction, infection/endotoxemia, and hyperfibrinolysis significantly contribute to platelet consumption. Among these, infection and endotoxemia represent the most urgent and modifiable targets.

## 3 Treatment

### 3.1 Treatment of Thrombocytopenia

Patients with cirrhosis and thrombocytopenia often suffer from delays in treatment or diagnosis due to bleeding risks. A platelet count ≥ 50 × 10^9^/L is commonly used as the intervention threshold, but the safe threshold depends on clinical judgment of bleeding risk [58].

#### 3.1.1 Platelet Transfusion

Platelet transfusion remains the most direct therapeutic measure for rapidly increasing the platelet counts; however, it is recommended only in the following situations: (1) critically ill patients at a high risk of bleeding and (2) patients requiring emergency high‐risk surgery or invasive procedures [4, 59]. Platelet transfusion is a transient solution, lasting only 48 h, and transfused platelets are rapidly cleared from the circulation [60]. Treatment also carries risks, including acute transfusion reactions, infection transmission, alloimmunization, and transfusion failure, limiting its use to short‐term or emergency situations [61].

#### 3.1.2 Spleen‐Targeted Therapies

Splenectomy is primarily indicated for patients with severe splenomegaly and pronounced hypersplenism (platelet count < 30 × 10^9^/L) and is often conducted in combination with shunt and devascularization procedures [59]. A retrospective study indicated that splenectomy significantly and sustainably improves thrombocytopenia in patients with cirrhosis and HCC, with effects lasting up to 3 years [62]. However, owing to the invasiveness and a considerable risk of complications, this procedure is recommended only for patients with well‐preserved liver function (Child–Pugh Class A, MELD score < 10) and without significant coagulopathy. Given the 10% incidence of portal vein thrombosis (PVT), patients should be vigilant against thrombotic risk postoperatively [63].

Partial splenic embolization (PSE) induces ischemia and necrosis in splenic tissue by selectively embolizing splenic artery branches to alleviate hypersplenism. The extent of embolization is crucial for efficacy. Long‐term studies have shown that in cirrhosis, platelet counts significantly increase after PSE, with higher increases correlated to the embolized area. When embolization covers less than 50%, therapeutic effects last only about 6 months [64]. Several studies supported this conclusion, showing a significant correlation between the proportion of splenic infarction and non‐infarcted volume with improved platelet counts [65, 66]. PSE is associated with various complications, including fever, abdominal pain, nausea, vomiting, post‐embolization syndrome, splenic abscess, pleural effusion, and ascites. Splenic infarction volume ≥ 540 mL (*p* = 0.031) and Child–Pugh Class C (*p* = 0.012) were found to be significantly correlated with the risk of complications [64, 66, 67]. Therefore, PSE is indicated for patients with Child–Pugh Class A or B cirrhosis whose thrombocytopenia is associated with splenomegaly or hypersplenism. In patients with Child–Pugh Class C cirrhosis, the risks and benefits should be carefully assessed before proceeding. A non‐randomized trial comparing total splenic artery embolization (TSAE) with PSE reported lower complication rates (*p* = 0.001), shorter hospital stays (*p* = 0.007), and higher long‐term postoperative platelet counts in the TSAE group (*p* = 0.001) [68]. TSAE may be more effective than PSE for treating thrombocytopenia, but larger studies are needed to validate this finding.

Splenic radiofrequency ablation (RFA) is a cost‐effective and minimally invasive procedure with a lower risk of surgical complications than other types of splenic surgery [69]. Studies have shown that platelet counts rise rapidly after RFA and gradually decline in patients with splenomegaly due to liver cirrhosis, returning to baseline levels by 48 months. This suggests the possible role of compensatory splenic hyperplasia. Patients with less than 50% splenic ablation volume are likely to experience splenomegaly recurrence at 6 months [70]. Studies have suggested that splenic ablation rates ≥ 40% may be correlated more strongly with improved outcomes of thrombocytopenia [71]. Nevertheless, the efficacy and long‐term stability of this treatment require validation through further clinical trials with longer follow‐ups.

Transjugular intrahepatic portosystemic shunt (TIPS) has shown significant variability in the treatment of thrombocytopenia. Although several retrospective studies have indicated short‐term improvements in platelet counts among patients with cirrhosis undergoing TIPS, long‐term follow‐up data suggest that platelet levels return to baseline within 12–14 months after the procedure [72, 73, 74]. Although TIPS can offer temporary relief by reducing portal pressure and alleviating splenic sequestration of blood cells, it does not address the underlying etiology of hypersplenism.

#### 3.1.3 Pharmacological Management

1. Recombinant human interleukin‐11 (rhIL‐11): As a megakaryocyte lineage enhancer, rhIL‐11 acts directly on megakaryocyte precursors rather than mature platelets [75]. In China, rhIL‐11 has been approved for treating Grades 3 and 4 thrombocytopenia following chemotherapy for solid tumors and nonmyeloid leukemias [4]. In a study of 10 patients with cirrhosis and thrombocytopenia, platelet counts increased after 10 days of rhIL‐11 treatment, reaching ≥ 60 × 10^9^/L [76]. In the aforementioned study, common adverse reactions included conjunctival hyperemia and edema, necessitating caution in patients with decompensated cirrhosis. A preliminary study in 20 HCV‐infected patients showed that after 12 weeks of daily rhIL‐11 treatment, the Knodell histology activity index (HAI) improved in 55% (*p* = 0.006) and platelet levels increased from 143 × 10^9^/L to 198 × 10^9^/L at Week 12 (*p* < 0.001), but all patients developed lower limb edema [77]. Thus, patients with cirrhosis should be closely monitored for ascites and systemic edema during treatment.
2. Recombinant human thrombopoietin (rhTPO): rhTPO mimics endogenous TPO and serves as an exogenous supplement for treating thrombocytopenia due to insufficient TPO synthesis in patients with CLD. A multicenter observational study found that among patients with hepatitis B‐related cirrhosis and thrombocytopenia, 73.3% achieved platelet counts greater than or equal to two times baseline after 1 month of treatment with rhTPO, and 54.8% achieved platelet counts greater than or equal to two times baseline after 6 months [78]. A prospective study of 70 patients with acute‐on‐chronic liver failure (ACLF) found that after 7 days of treatment with rhTPO, 60.7% of patients achieved platelet counts > 50 × 10^9^/L by Day 14, compared to 12% in the control group (*p* < 0.001) [79]. Available clinical evidence indicates that rhTPO effectively increases platelet counts in patients with CLD and reduces the need for platelet transfusions, but its long‐term safety needs further assessment [78, 80].
3. TPO‐RAs: TPO‐RAs mimic endogenous TPO by binding to and activating TPO receptors on megakaryocytes, promoting their proliferation and differentiation to platelets. Patients whose thrombocytopenia is predominantly driven by insufficient TPO production respond well to TPO‐RAs, which are suitable for short‐term platelet count elevation before elective invasive procedures.
  ○ Eltrombopag: Eltrombopag is an oral, small‐molecule, non‐peptide TPO‐RA. It activates the transmembrane domain of the TPO receptor, promoting bone marrow progenitor cell proliferation and differentiation and enhancing platelet production [81]. A Japanese Phase II trial with 38 patients with CLD showed that eltrombopag increases platelet counts in a dose‐dependent manner, but higher doses may lead to severe adverse events [82]. A multicenter randomized Phase II study confirmed that eltrombopag significantly and dose‐dependently increases platelet counts in patients with hepatitis C‐related cirrhosis. By Week 4, 75%–95% of patients achieved platelet counts ≥ 100 × 10^9^/L (*p* < 0.001) without serious adverse events [83]. Studies have indicated that eltrombopag can significantly reduce the need for perioperative platelet transfusion (72% vs. 19%, *p* < 0.001). This study found a higher incidence of thrombotic events in the treatment group, resulting in the early termination of the trial [84]. Two international Phase III trials showed that treatment with eltrombopag helped more patients with HCV and thrombocytopenia initiate and sustain their antiviral therapy, resulting in higher sustained virological response rates (23% vs. 14%, *p* = 0.0064; 19% vs. 13%, *p* = 0.0202). However, the treatment group had higher rates of hepatic decompensation and thromboembolic events, which warrant high clinical vigilance [85].
  ○ Avatrombopag: Avatrombopag is an orally bioavailable small‐molecule TPO‐RA. A study of 130 patients with cirrhosis and thrombocytopenia found that treatment with avatrombopag 1 week before elective surgery achieved target rates of 49% and 47.6% in two dose groups, significantly higher than the placebo group (6.3% and 9.5%). Common side effects included nausea, fatigue, and headache, with overall good tolerability [86]. In the phase III trial of avatrombopag, more patients in the treatment group achieved a preoperative platelet count of ≥ 50 × 10^9^/L and did not need platelet transfusions compared to the placebo group. There was no significant difference between the two groups in terms of the incidence or severity of adverse events [87]. A subgroup analysis showed that avatrombopag increased platelet counts in patients with CLD without increasing platelet activation or reactivity or elevating the risk of adverse events [88].
  ○ Lusutrombopag: Lusutrombopag is an orally administered synthetic small molecule. In a phase III trial comprising 96 patients with CLD and severe thrombocytopenia, a significantly higher proportion of patients treated with lusutrombopag avoided platelet transfusions before invasive procedures compared to the placebo group (79.2% vs. 12.5%, *p* < 0.0001). Among lusutrombopag‐treated patients, the median platelet counts exceeded 50 × 10^9^/L after 5 days, with a median time to peak of 13.4 days [89]. Another global multicenter randomized double‐blind Phase III trial enrolled 215 patients with CLD and baseline platelet counts < 50 × 10^9^/L to assess the therapeutic effects of lusutrombopag [90]. In the lusutrombopag group, 64.8% (70/108) of patients achieved the primary endpoint, which was significantly higher than 29.0% (31/107) in the placebo group (*p* < 0.0001). In addition, the median platelet count maintenance was 19.2 days at ≥ 50 × 10^9^/L. Adverse event rates were similar between groups, being mostly mild to moderate. Only one thromboembolic event occurred in the lusutrombopag group, with no clear link to platelet count. Subsequent studies supported the efficacy and safety of lusutrombopag treatment for CLD‐associated thrombocytopenia [91, 92].
  ○ Romiplostim: Romiplostim is a recombinant protein with a peptide chain fused to the IgG1 Fc domain, lacking homology to endogenous TPO. Romiplostim exhibited high clinical efficacy and good tolerability in patients with chronic immune thrombocytopenia (ITP) [93]. Romiplostim shows promise for treating CLD‐associated thrombocytopenia due to its efficacy in chronic ITP. In a study including 35 patients with hepatitis C‐related cirrhosis and thrombocytopenia, romiplostim increased platelet counts to ≥ 70 × 10^9^/L in 33 patients (94.3%), making them eligible for surgery. Of these, 20% sustained platelet counts > 50 × 10^9^/L 3 months posttreatment; however, its safety should be further validated [94] (Table 1).

**TABLE 1:** Studies of thrombopoietic agents for CLD‐related thrombocytopenia.

Medicine	Study type	Sample	Method	Curative effect	Adverse reaction
rhIL‐11	Single‐center, non‐randomized prospective trial [76]	*n* = 10, HCV‐induced cirrhosis, PLT < 80 × 10^9^/L	50 μg/kg/day for 10 days	Increase PLT above the baseline value, *p* < 0.01.	Fluid retention, red eyes, joint ache, headache
	Open‐label study [77]	*n* = 20, HCV‐induced cirrhosis	5 μg/kg/day for 12 weeks	PLT increased from a mean of 143 × 10^9^/L to 198 × 10^9^/L, *p* < 0.001.	Edema of lower extremity
rhTPO	Multicenter real‐world observational study [78]	*n* = 126, HBV‐induced cirrhosis, PLT < 30 × 10^9^/L	15 000 U/day for 14 days	PLT is higher in rhTPO group.	Infection, weight gain, headache, dyspepsia, nausea, pyrexia, and dizziness
	Prospective, open‐label study [79]	*n* = 70, ACLF, PLT < 50 × 10^9^/L	15 000 U/day for 7 days	PLT > 50 × 10^9^/L was 60.7%, *p* < 0.001.	Transient fever
Eltrombopag	Multicenter randomized, open‐label, phase 2 study [82]	*n* = 38, CLD, Child–Pugh Class A or B, PLT < 50 × 10^9^/L	12 pts, 12.5 mg/day; 14 pts, 25 mg/day; 12 pts, 37.5 mg/day; all for 2 weeks	PLTs were 24.8 × 10^9^/L, 54 × 10^9^/L, and 60 × 10^9^/L in the 12.5, 25, and 37.5 mg groups.	One ascites, one pleural effusion, and portal vein thrombosis 22 days posttreatment in the 37.5 mg group
	Multicenter, double‐blind, randomized, placebo‐controlled, phase 2 trial [83]	*n* = 74, HCV‐induced cirrhosis, PLT 20–70 × 10^9^/L	18 pts, placebo; 14 pts, 30 mg/day; 19 pts, 50 mg/day; 23 pts, 75 mg/day; all for 4 weeks	PLTs were increased to 100 × 10^9^/L or more in pts: 0 in placebo, 75% in 30 mg, 79% in 50 mg, 95% in 75 mg.	Headache, dry mouth, abdominal pain, and nausea
	Multicenter, double‐blind, randomized, placebo‐controlled, phase 3 trial [84]	*n* = 292, CLD, PLT < 50 × 10^9^/L	75 mg/day, or placebo for 14 days	59% pts has PLT more than 80 × 10^9^/L in the eltrombopag group.	Headache, pyrexia, abdominal pain, diarrhea, nausea, portal vein thrombosis
	Multicenter randomized, placebo‐controlled, phase 3 trial [85]	*n* = 715 (Group 1), 805 (Group 2), HCV‐induced cirrhosis, PLT < 75 × 10^9^/L	25–100 mg/day for 9 weeks or fewer	94% (Group 1), 95% (Group 2) pts reached the minimal platelet threshold. PLT ≥ 50 × 10^9^/L throughout treatment (eltrombopag vs. placebo: group 1, 69% vs. 15%; group 2, 81% vs. 23%).	Hepatic decompensation, thromboembolic events
Avatrombopag	Multicenter, randomized, placebo‐controlled, double‐blind, Phase 2 study [86]	*n* = 130, cirrhosis, PLT 10–58 × 10^9^/L	Cohort A: 100 mg loading dose followed by 20, 40, or 80 mg/day on Days 2–7. Cohort B: 80 mg loading dose followed by 10 mg/day for Days 2–7, or 20 mg/day for Days 2–4	The proportion of responders in the avatrombopag group is 48.4% vs. 8.1% in the placebo group, and shows a clear dose‐dependent response.	Nausea, fatigue, headache
	Multicenter, randomized, placebo‐controlled, double‐blind, Phase 3 trial [87]	*n* = 231 (ADAPT‐1), *n* = 204 (ADAPT‐2), CLD, PLT < 50 × 10^9^/L	60 mg/day or 40 mg/day for 5 days	Reach the primary endpoint: ADAPT‐1: 65.6% in 60 mg vs. 22.9% in placebo; 88.1% in 40 mg vs. 38.2% in placebo; ADAPT‐2: 68.6% in 60 mg vs. 34.9% in placebo; 87.9% in 40 mg vs. 33.3% in placebo.	Abdominal pain, dyspepsia, nausea, pyrexia, dizziness, headache
Lusutrombopag	Multicenter, randomized, double‐blind, placebo‐controlled, phase 3 study [89]	*n* = 96, CLD, PLT < 50 × 10^9^/L	3 mg/day for 7 days	The proportions of pts avoiding preoperative platelet transfusion: 79.2% (lusutrombopag) vs. 12.5% (placebo).	Nausea, pyrexia, headache, pain, portal vein thrombosis
	Global, randomized, double‐blind, placebo‐controlled, Phase 3 study [90]	*n* = 215, CLD, PLT < 50 × 10^9^/L	3 mg/day for ≤ 7 days	Avoiding preprocedure platelet transfusion: 64.8% (lusutrombopag) vs. 29.0% (placebo).	Headache, abdominal pain, fatigue, peripheral edema, nausea
Romiplostim	Single‐center, single‐arm, open‐label study [94]	*n* = 35, HCV‐induced cirrhosis, PLT < 50 × 10^9^/L	2 μg/kg Q1W for a maximum of 4 weeks or until two consecutive PLT of 70 × 10^9^/L	Achieving PLT of 70 × 10^9^/L: 94.3%.	No serious adverse events were observed

Abbreviations: ACLF, acute‐on‐chronic liver failure; CLD, chronic liver disease; HBV, hepatitis B virus; HCV, hepatitis C virus; PLT, platelet count; pts, patients; rhIL‐11, recombinant human interleukin‐11; rhTPO, recombinant human thrombopoietin.

Thrombotic events, particularly PVT, are the primary safety concerns associated with the use of TPO‐RAs. A meta‐analysis indicated that TPO‐RAs did not significantly increase the overall risk of PVT in patients with CLD; however, subgroup analyses revealed a significant association between the use of eltrombopag and the incidence of PVT [95]. Moreover, eltrombopag may increase the risk of liver decompensation and even lead to severe complications, such as acute liver failure and hepatic encephalopathy [85, 96]. In contrast, newer oral TPO‐RAs, such as avatrombopag and lusutrombopag, have shown a significantly lower thrombotic risk and no significant hepatotoxicity across multiple global studies. Therefore, avatrombopag and lusutrombopag are indicated for adult patients with CLD who are scheduled to undergo elective invasive procedures and meet the following criteria: (1) baseline platelet count < 50 × 10^9^/L and (2) absence of active bleeding [59, 97]. It is crucial to emphasize that TPO‐RAs primarily aim to raise platelet counts to a safe level sufficient to permit the procedure, rather than normalizing platelet counts. Therefore, treatment should be limited to short‐term use to avoid potential thrombotic events associated with long‐term maintenance therapy.

In patients with cirrhosis, the incidence of PVT substantially increases with worsening liver dysfunction and represents one of the common complications [98, 99]. Given the inherent thrombotic potential of all TPO‐RAs, stringent patient selection is required in clinical practice. TPO‐RAs should be contraindicated in patients with a current or previous thromboembolic event. Since the onset of their effects needs 5–7 days, TPO‐RAs are not suitable for emergency procedures. Patients with a MELD score > 20 should be prioritized for liver transplant evaluation. A careful risk–benefit assessment balancing bleeding and thrombotic risks is essential for patients with HCC or Child–Pugh Class C cirrhosis, and it should be undertaken with caution and enhanced monitoring if treatment is pursued [59, 97].

**FIGURE 1:**
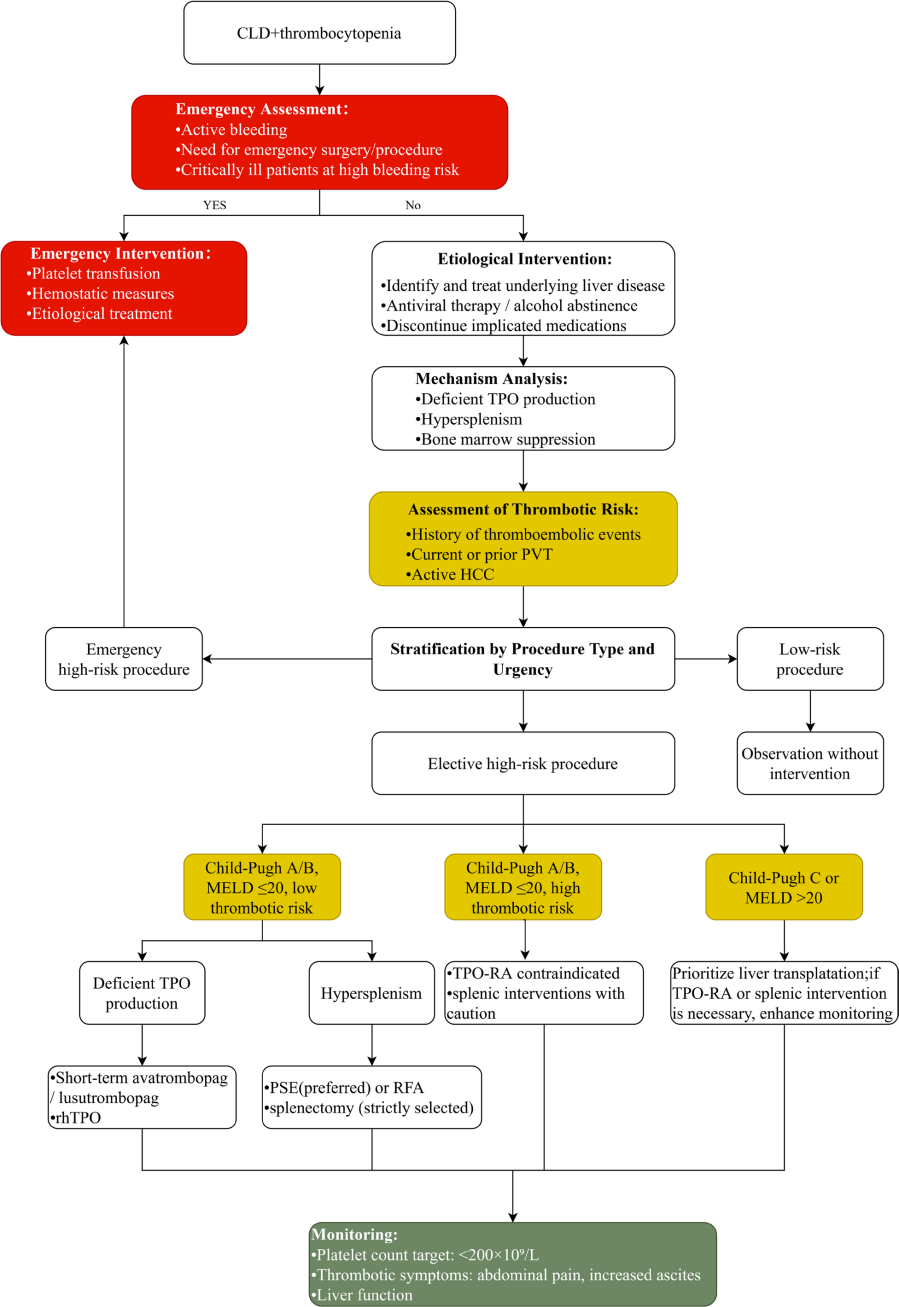
Flow chart of treatment of CLD‐related thrombocytopenia. High thrombotic risk: presence of any of the following: history of thromboembolic events; current or prior PVT; active HCC. Low thrombotic risk: absence of all of the following: history of thromboembolic events; current or prior PVT; active HCC. CLD, chronic liver disease; HCC, hepatocellular carcinoma; MELD, model for end‐stage liver disease; PSE, partial splenic embolization; PVT, portal vein thrombosis; RFA, radiofrequency ablation; rhTPO, recombinant human thrombopoietin; TPO, thrombopoietin; TPO‐RA, thrombopoietin receptor agonist.

All patients should undergo imaging to exclude PVT before treatment initiation. Moreover, close monitoring of platelet counts, thrombotic symptoms (such as abdominal pain and increased ascites), and liver function is required during treatment [97]. Evidence suggests that using lower TPO‐RA doses and targeting a platelet count of < 200 × 10^9^/L may help reduce the risk of PVT [95, 100]. For patients with a history of PVT, advanced HCC, or significant thrombotic risk, alternative strategies should be considered, or more intensive monitoring should be implemented. Clinical decision‐making should balance the risk of bleeding against thrombosis, with careful consideration of hepatic dysfunction severity and procedural characteristics to guide the development of individualized treatment strategies (Figure 1).

### 3.2 Treatment of Leukopenia

Studies have indicated that spontaneous bacterial peritonitis constitutes 36% of infections in CLD, followed by urinary tract infections (22%), pneumonia (19%), and skin/soft tissue infections (8%) [101]. Patients with cirrhosis often present with leukopenia, particularly neutropenia, increasing the risk of infection‐related mortality.

#### 3.2.1 Hematopoietic Growth Factors

Hematopoietic growth factors are glycoproteins that promote the proliferation and differentiation of bone marrow progenitor cells. G‐CSF, GM‐CSF, macrophage colony‐stimulating factor (M‐CSF), and interleukin‐3 (IL‐3) are the four main myeloid growth factors used in clinical practice [102, 103]. GM‐CSF increases white blood cell and neutrophil counts in cirrhosis complicated by hypersplenism [20]. The efficacy of G‐CSF is well recognized in the treatment of neutropenia, significantly improving patients' prognosis and quality of life [104, 105].

G‐CSF is used for both prophylactic and therapeutic purposes in clinical practice. Myelosuppression induced by antineoplastic agents and other treatments can lead to severe complications, such as febrile neutropenia (FN), resulting in treatment delays and poor prognosis. Prophylactic use of G‐CSF is a core strategy for lowering the risk of FN, particularly for patients receiving chemotherapy regimens associated with a high risk of FN [106]. G‐CSF should be used based on the severity of neutropenia and infection risk. Since patients with cirrhosis are already at a high risk of infection, the presence of neutropenia significantly increases this risk, necessitating proactive interventions [7]. Among patients with cirrhosis, mild neutropenia, and no infection, prophylactic G‐CSF may be considered in selected high‐risk patients undergoing chemotherapy or invasive procedures. Therapeutic administration is typically indicated for patients with cirrhosis and moderate to severe neutropenia (neutrophil count < 1.0 × 10^9^/L), particularly in the presence of active or recurrent infections.

Recombinant human G‐CSF (rhG‐CSF) has a short half‐life (nearly 4–8 h) and requires daily administration until neutrophil recovery [104, 107]. In contrast, due to its significantly extended half‐life, long‐acting polyethylene glycol‐conjugated rhG‐CSF (PEG‐rhG‐CSF) enables single‐dose administration per chemotherapy cycle [108]. Previous studies have mainly addressed chemotherapy‐induced neutropenia, but its effects on patients with CLD have no significant difference, and PEG‐rhG‐CSF can improve patient compliance [109]. Available evidence does not confirm the risks of myeloid malignancies in adults with chronic neutropenia, requiring long‐term treatment with growth factors. The most common adverse effect of growth factors is mild to moderate bone pain, with no significant difference between the two types of formulations. Other frequent reactions include headache, myalgia, and fatigue [110].

G‐CSF should be used cautiously in patients with advanced liver disease and ACLF. Early small‐scale studies reported that G‐CSF may improve survival rates in patients with decompensated cirrhosis and ACLF [111]. However, subsequent large‐scale randomized controlled trials failed to confirm the survival benefits of G‐CSF in ACLF; showed no improvement in liver function or infection rates; and reported more adverse events [112, 113]. Notably, the number of patients experiencing ACLF during follow‐up was approximately twice as high in the G‐CSF group as in the placebo group. Among seven cases experiencing serious adverse events, five cases were associated with worsening organ dysfunction. Studies have shown that in an alcohol‐induced mouse model of ACLF, G‐CSF induced liver regeneration and neutrophil infiltration, but the infiltrating neutrophils exhibited an activated phenotype characterized by increased expression of CXC motif chemokine receptor 2, leukotriene B4 receptor 1, and calprotectin [114]. This finding suggests that G‐CSF may exacerbate liver damage in ACLF by promoting neutrophil infiltration and calmodulin expression. A study combining G‐CSF with the Toll‐like receptor‐4 inhibitor TAK‐242 in a mouse model of ACLF revealed that this therapeutic regimen significantly improved liver function, suppressed inflammatory responses, promoted liver regeneration, and reduced mortality [115]. In summary, the use of G‐CSF in patients with decompensated cirrhosis and ACLF requires careful consideration of potential extrahepatic organ damage due to its pro‐inflammatory effects.

#### 3.2.2 Interleukin‐19 (IL‐19)

Osteoblasts, key regulatory cells in the hematopoietic microenvironment, secrete IL‐19, which promotes granulopoiesis and neutrophil production. However, the role and mechanisms of this cytokine in neutrophil development remain unclear. Clinical studies have shown that low‐dose IL‐19 can effectively improve radiation therapy, chemotherapy, or drug‐induced neutropenia in mouse models [116]. The potential efficacy and safety of IL‐19 in CLD‐associated neutropenia are still unclear in preclinical research.

#### 3.2.3 Spleen‐Targeted Therapies

Spleen‐targeted treatments can improve white blood cell counts in leukopenia with hypersplenism. Laparoscopic splenectomy significantly increased leukocyte counts postoperatively compared to preoperative levels (median: 9.47 vs. 2.68, *p* < 0.001) in patients with cirrhosis [117]. White blood cell counts exhibit a significant short‐term increase in patients undergoing RFA, which gradually declines over time [70, 71]. PSE effectively increased leukocyte counts when treating secondary hypersplenism in CLD [118, 119]. In a long‐term follow‐up study, the leukocyte counts of patients with cirrhosis and ≥ 50% splenic embolization coverage remained significantly higher than preoperative levels in 5 years postoperatively (*p* < 0.05). When embolization coverage exceeded 70%, the risk of severe complications significantly increased. It is recommended to keep splenic infarction rates between 50% and 70% to ensure therapeutic efficacy and prevent complications [64].

#### 3.2.4 Other Treatments

Patients with cirrhosis and neutropenia should be vigilant for bacterial infections if they develop fever, chills, or localized symptoms. In case of sepsis, septic shock, or ANC < 0.5 × 10^9^/L, empirical broad‐spectrum antibiotics should be started immediately after obtaining culture specimens [120, 121]. After 48 h of treatment, the decision regarding adjusting the treatment regimen should be made based on the type of bacterial infection and the initial response to treatment [120]. Studies have shown that approximately 20% of patients with cirrhosis and septic shock receive inappropriate initial antibiotics, reducing survival rates by up to fivefold. Furthermore, each hour of delay in initiating appropriate antibiotics increases the risk of mortality by approximately 10% [122, 123]. A clinical trial confirmed that Licojun tablets can effectively prevent and treat leukopenia, with good safety profiles [124]. Currently, Licojun tablets are used as an adjunctive therapy for cirrhosis‐associated leukopenia.

**FIGURE 2:**
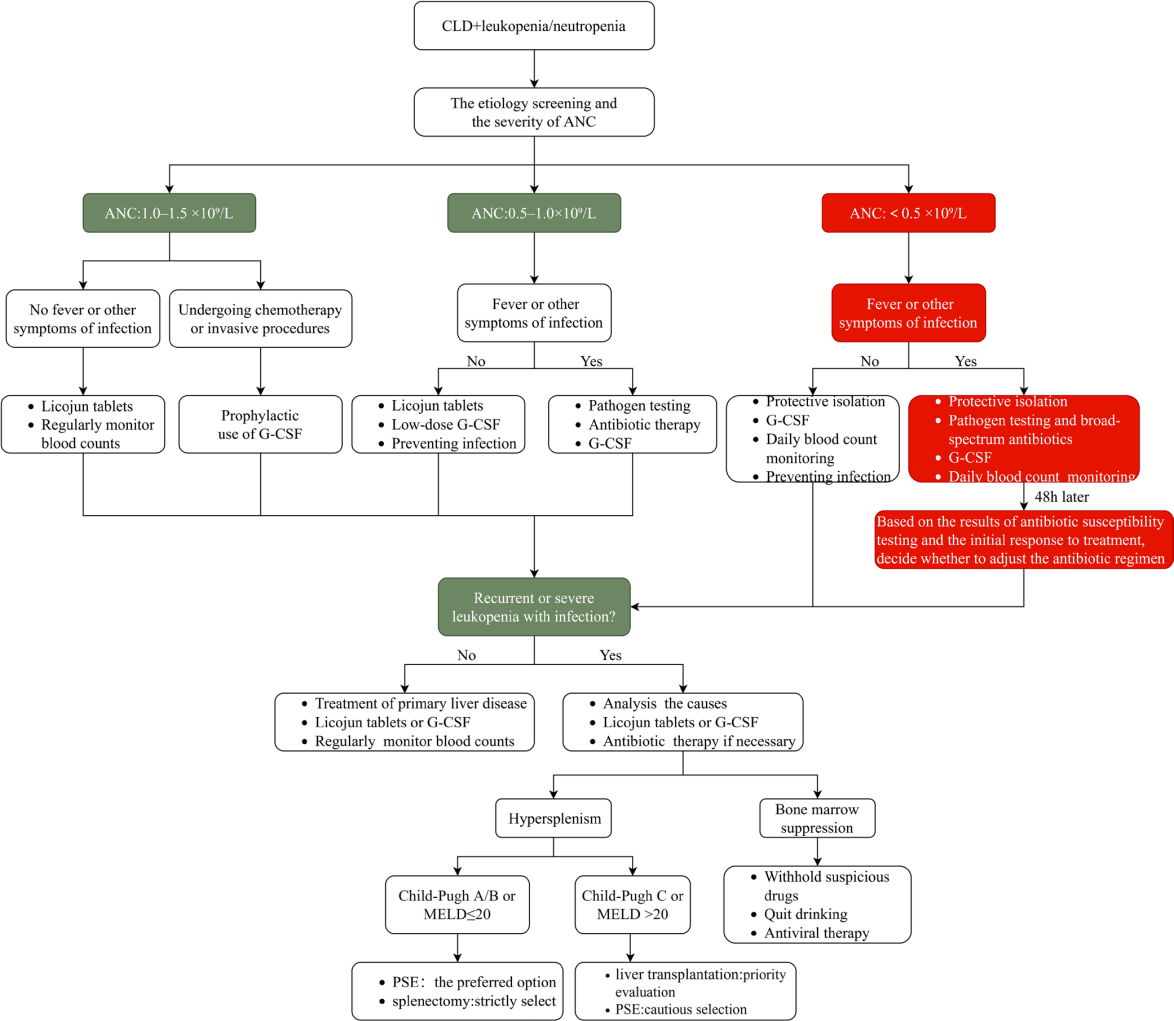
Flow chart of treatment of CLD‐related leukopenia. ANC, absolute neutrophil count; CLD, chronic liver disease; G‐CSF, granulocyte colony‐stimulating factor; MELD, model for end‐stage liver disease; PSE, partial splenic embolization.

In patients with CLD presenting with recurrent or severe leukopenia, the underlying mechanism should be elucidated, and etiology‐directed interventions should be implemented. PSE or splenectomy may be considered if leukopenia is predominantly attributed to hypersplenism, but these interventions should be reserved for those with Child–Pugh Class A or B. Liver transplantation should be prioritized for patients with Child–Pugh Class C or MELD score > 20, and PSE should be used with caution. When leukopenia primarily originates from bone marrow suppression, the cornerstone of management is the removal of causative factors, including discontinuation of implicated medications, antiviral therapy, and alcohol abstinence (Figure 2).

## 4 Conclusion

Thrombocytopenia often coexists with leukopenia, and they are usually accompanied by anemia and coagulation abnormalities. Hypersplenism is a major contributor to both types of cytopenia. Thrombocytopenia primarily arises from inadequate TPO synthesis, while bone marrow suppression can originate from viral infections, alcohol abuse, or medications. Identification of the main pathological mechanism is crucial for developing targeted treatment strategies.

Treatment options should be evaluated for efficacy, safety, invasiveness, cost, and patient preference. Platelet transfusions can rapidly raise platelet counts but offer only short‐term support in emergency situations or before invasive procedures. Spleen‐targeted therapies provide more sustained improvements in blood cell counts but carry risks such as surgical trauma, PVT, postoperative pain, and infection. The effectiveness and complication rates of PSE rely on the extent of embolization. TIPS can also reduce portal vein pressure and improve cytopenia. Available studies suggest that the combination of TIPS and PSE may be superior to monotherapy in reducing variceal rebleeding and maintaining shunt patency [125, 126]. In patients with cirrhosis, TPO‐RAs, such as avatrombopag and lusutrombopag, represent effective options for increasing platelet counts before elective procedures; however, rigorous assessment of thrombotic risk is required when using these agents. Thrombocytopenia is only one factor affecting bleeding risk in patients with cirrhosis. The hemostatic system of patients with cirrhosis is in a unique “rebalancing” state, with complex changes in coagulation factors, platelet function, and fibrinolytic activity. Some patients may show hypercoagulable tendencies [127]. Therefore, bleeding risk assessment requires a comprehensive evaluation of multiple indicators, including platelet count, clinical presentation, and coagulation function [128]. Enhanced monitoring and management of thrombotic risk are essential when using TPO‐RAs to boost platelet counts.

Although earlier studies indicated that G‐CSF may improve survival in patients with ACLF, subsequent studies have not confirmed a survival benefit. Instead, recent studies observed an increased incidence of adverse events along with pro‐inflammatory effects, suggesting that the use of G‐CSF in patients with ACLF requires great caution.

Despite advances in understanding cytopenia in cirrhosis, challenges remain. Herein, optimal treatment strategies for patients with varying Child–Pugh grades and etiologies remain unclear. Furthermore, the long‐term effects of TPO‐RAs on HCC and thrombotic events, as well as the optimal dosing and duration of treatment, need further studies. Moreover, sequential or combined treatment regimens require optimization through high‐quality studies. Future studies should explore disease mechanisms, refine treatment strategies, and develop novel treatment options to improve long‐term quality of life for patients with cirrhosis.

## Funding

This work was supported by the Construction Funding for the Key Specialty of Integrated Traditional Chinese and Western Medicine in Liver Diseases (2A62091). The Class B of Renhe Academy (2D12650).

## Ethics Statement

The authors have nothing to report.

## Consent

The authors have nothing to report.

## Conflicts of Interest

The authors declare no conflicts of interest.

## Data Availability

The authors have nothing to report.
